# Cancer-associated fibroblasts promote malignant phenotypes of prostate cancer cells via autophagy: Cancer-associated fibroblasts promote prostate cancer development

**DOI:** 10.1007/s10495-023-01828-2

**Published:** 2023-03-31

**Authors:** XuKai Liu, JiZu Tang, LiQiang Peng, HaiBo Nie, YuanGuang Zhang, Pan Liu

**Affiliations:** 1 Department of Neurosurgery, Zhuzhou Central Hospital, Zhuzhou, Hunan Province 412007 P.R. China; 2 Department of Orthopaedics, Zhuzhou Central Hospital, Zhuzhou, Hunan Province 412007 P.R. China; 3 Department of Trauma Center, Zhuzhou Central Hospital, Zhuzhou, Hunan Province 412007 P.R. China; 4 Department of Urology, Zhuzhou Central Hospital, Zhuzhou, Hunan Province 412007 P.R. China; 5 Department of Spine surgery, Zhuzhou Central Hospital, No. 116, Changjiang South Road, Tianyuan District, Zhuzhou, Hunan Province 412007 P.R. China; 6 Department of Emergency, Zhuzhou Central Hospital, No. 116, Changjiang South Road, Tianyuan District, Zhuzhou, Hunan Province 412007 P.R. China

**Keywords:** Cancer-associated fibroblasts, ATG5, Autophagy, Prostate cancer, Malignant phenotype

## Abstract

Dysregulation of autophagy in cancer-associated fibroblasts (CAFs) has been demonstrated to play a role in malignant phenotypes of human tumors. We intended to investigate the function of CAFs autophagy in prostate cancer (PCa). Firstly, CAFs and normal fibroblasts (NFs) were isolated from cancerous and adjacent normal tissues of PCa patients, for the following experimental preparation. In comparison with NFs, CAFs expressed higher levels of the myofibroblast marker ?-smooth muscle actin (?-SMA) and the mesenchymal marker Vimentin. Besides, CAFs possessed a higher autophagic level than NFs. As for malignant phenotypes, PCa cells co-cultured with CAFs-CM showed greater proliferation, migration and invasion capabilities, while these outcomes were obviously abolished by autophagy inhibition with 3-Methyladenine (3-MA). Moreover, silencing of ATG5 in CAFs inhibited fibroblasts autophagic level and suppressed malignant phenotypes of PCa cells, while ATG5 overexpression in NFs exerted opposite effects. Depletion of ATG5 in CAFs inhibited the xenograft tumor growth and lung metastasis of PCa cells. Taken together, our data demonstrated the promotive effect of CAFs on PCa malignant phenotypes through ATG5-dependent autophagy, suggesting a novel mechanism for PCa progression.

**Supplementary Information:**

The online version contains supplementary material available at 10.1007/s10495-023-01828-2.

## Introduction

Prostate cancer (PCa) is a common malignancy [1] and a major cause of cancer-related death in men worldwide [2]. Currently, approaches for the treatment of PCa has evolved significantly and therapy options for patients includes hormonal therapy, chemotherapy, immunotherapy and targeted therapy [3]. Despite the innovations in treatments have improved the outcomes, metastatic PCa is still related with considerably diminished overall survival [4]. Therefore, elucidating the mechanism of PCa metastasis is of great importance for ameliorating the prognosis of patients.

Recently, numerous studies have demonstrated the critical role of tumor microenvironment (TME) in the development of tumors [5]. Survival, growth and migration of cancer cells are influenced by the surrounding TME [6]. Among the components of TME, cancer-associated fibroblasts (CAFs) are the majority ones of stromal cells [7]. Compared to normal fibroblasts (NFs), CAFs exhibit higher expressions of specific markers, including α-smooth muscle actin (α-SMA) [8]. CAFs are reported to be implicated in the development of various types of cancers, such as lung cancer [9], gastrointestinal cancer [10] and PCa [11]. Prostate CAFs often express androgen receptor and are sensitive to androgens [12]. Moreover, they are key players in metabolic changes that drive prostate tumor growth [13].

Autophagy, an intracellular degradation process, is able to degrade intracellular components such as macromolecular complexes, organelles and proteins [14]. It is a core molecular pathway for the maintenance of organismal and cellular homeostasis [15] and implicates in cancer proliferation, invasion and metastasis [16]. Recent studies also reveal that autophagy plays a vital role in the functions of CAFs, for instance, CAFs secretes cytokines to promote cancer survival by autophagy [17]. Hence, targeting autophagy in CAFs with pharmacological inhibitors or inducers has gained importance in preclinical trials [18]. Nevertheless, whether CAFs autophagy affects PCa development remains to be explored.

Autophagy is mediated by evolutionarily conserved autophagy-related (ATG) genes [19]. ATG proteins are involved in autophagy initiation, maturation and degradation and involved in cancer progression [20]. Among that, autophagy-related 5 (ATG5) is considered to be an essential molecule for the induction of autophagy [21]. ATG5-mediated autophagy acts as a key mechanism that controls the growth and metastasis of PCa [22, 23]. Furthermore, in non-small cell lung cancer, cell invasion can be inhibited dramatically when CAFs autophagy is repressed by silencing of ATG5 [24]. However, whether the level of ATG5 in CAFs affects PCa development is undetermined.

In this research, we sought to examine the role of PCa-derived CAFs in PCa progression and explore the potential mechanisms. We found that PCa-derived CAFs highly expressed ATG5 and exhibited high basal level of autophagy, which facilitated PCa growth and metastasis.

## Materials and methods

### Clinical samples

In this study, PCa patients (n = 5) receiving radical prostatectomy from Zhuzhou Central Hospital were recruited. They accepted no radiotherapy or chemotherapy before the surgery. The adjacent normal tissues (> 3–5 cm away from tumor) and cancerous tissues were resected surgically. Two clinical pathologists were responsible for validating the tissue specimens. Clinicopathological information was presented in Table S2. This research was approved by Ethics Committee in Zhuzhou Central Hospital and written consents were acquired from the patients.

### Isolation and culture of CAFs and NFs

NFs and CAFs were isolated from adjacent normal tissues and PCa specimens, respectively. In brief, after rinsed by sterile phosphate-buffered saline (PBS), samples were mechanically minced into small pieces (1 mm^3^). In a humidified environment with 5% CO_2_ and 95% air at 37 °C, they were digested in 0.12% collagenase A for 8 h. Then the digestion was stopped by the supplementation of Dulbecco’s modified Eagle’s medium (DMEM, Thermo, Waltham, MA, USA) plus 10% fetal bovine serum (FBS, Thermo). After removed the tissue debris, cells were gathered and maintained in a humidified environment with 5% CO_2_ and 95% air at 37 °C. Cells were reseeded when they reached 80% confluence. Fibroblasts ranging from passage 2–6 were used in this study in accordance with the previous research [25].

For the treatment of autophagy inhibitor 3-Methyladenine (3-MA, Sigma, St. Louis, MO, USA), 5 mM of 3-MA was added into CAFs and incubated for 24 h. To prepare the conditioned medium (CM), CAFs or NFs separated from tissue samples were first maintained in DMEM containing 10% FBS for 72 h. Next, the supernatants were gathered, followed by centrifuged for 15 min at 1200 rpm for removing cell debris and obtaining the corresponding CM. Finally, PCa cells were incubated with CM collected from CAFs or NFs for 24 h.

### Cell culture

PC-3 and DU145 PCa cell lines were acquired from American Type Culture Collection (Manassas, VA, USA). Under a humidified environment at 37 °C with 5% CO_2_, cells were incubated with DMEM supplemented with 1% penicillin/streptomycin and 10% FBS.

### Cell transfection

Smaller interfering RNA (siRNA) against ATG5 (si-ATG5-1, si-ATG5-2 and si-ATG5-3), negative control (si-NC), empty vector pcDNA3.1 and overexpression plasmid of pcDNA3.1-ATG5 were obtained from Gene Pharma (Shanghai, China). Afterwards, cells were planted in six-well plates and cultured overnight. Next, according to the manufacturer’s guidelines, the above oligonucleotides were transfected into cells with Lipofectamine 2000 (Thermo) for 48 h. Cells were collected for the following experiments.

### Immunofluorescence

After fixed by 4% paraformaldehyde (Beyotime, Jiangsu, China), cells were incubated in 0.25% Triton X-100 and 1% bovine serum albumin (BSA) in PBS. Then, they were incubated in primary antibodies, including anti-Vimentin (Abcam, Cambridge, MA, USA), anti-α-SMA (Sigma) and anti-LC3B (Abcam), overnight at 4 °C. Following rinsed in PBS, they were incubated in fluorescein-conjugated secondary antibodies at room temperature for 1 h. 4,6-diamidino-2-phenylindole (DAPI) was applied for nuclei staining and a fluorescence microscope (Olympus, Tokyo, Japan) was used for acquiring fluorescence images. LC3B puncta per cell were calculated with ImageJ.

### Cell counting Kit-8 (CCK-8) assay

A CCK-8 kit (Beyotime) was applied to assess cell proliferation. PCa cells (2 × 10^3^ cells/well) were planted in 96-well plates. Following incubation overnight, cells were treated with the corresponding reagents for 24, 48, 72 and 96 h. Each well was added with CCK-8 solution (10 µl, Beyotime) before incubation for 2 h at 37 °C. A microplate reader (Bio-Rad, Hercules, CA, USA) was applied to record the absorbance of each sample at 450 nm.

### Wound healing assay

Cells (2.0 × 10^5^ cells/well) were seeded in six-well plates until 80% confluent. Following the indicated treatment, 10 µl-pipette tip was used to scratch the monolayer cells. Then PBS was applied to rinse the cells for removing the non-adherent cells. After 12 h incubation, cells were rinsed with PBS. A microscope (Olympus) was employed to take the pictures at 0 and 12 h. The wounded area (%) was calculated: (wound area at 0 h-wound area at 12 h)/wound area at 0 h × 100 (%).

### Transwell assay

For invasion assay, Transwell inserts (8 µM pore size, BD Biosciences, San Jose, CA, USA) were utilized. Briefly, PCa cells (5 × 10^5^ cells/mL) were resuspended in serum-free medium. Then the upper chamber coated with Matrigel was added with 200 µL cell suspension. The medium containing 10% FBS was added into the lower chamber. Cells were routinely cultured for 48 h. Following fixed for 30 min with 4% paraformaldehyde, the invaded cells were stained using 0.5% crystal violet (Sigma). Cell numbers were counted in three random fields under a microscope.

### RT-qPCR

Total RNA of tissues and cells was obtained with Trizol reagent (Takara, Tokyo, Japan). The cDNA was synthesized with the total RNA using RNA reverse transcription kit (Takara). For gene expression analysis, RT-qPCR was operated with Taqman PCR master mix (Applied Biosystems, Foster City, CA, USA) applying the StepOnePlus real-time PCR System (Applied Biosystems). Table S1 showed the primer sequences. The relative mRNA level was determined applying 2^ΔΔCT^ method. GAPDH was employed as an internal control.

### Western blot

Total proteins of tissues and cells were isolated using RIPA lysis buffer (Beyotime). Equal amounts of cell lysate were electrophoresed on SDS-polyacrylamide gels, then proteins were removed to polyvinylidene difluoride membranes. After blocked with 5% nonfat milk for 1 h, membranes were incubated in primary antibodies at 4 ◦C overnight. Primary antibodies included anti-Vimentin (Abcam), anti-α-SMA (Sigma), anti-Beclin1 (Abcam), anti-LC3 I/II (Invitrogen, Carlsbad, CA, USA), anti-p62 (Abcam), anti-β-tubulin (Abcam) and anti-ATG5 (Abcam). Then membranes were incubated in HRP-conjugated secondary antibodies at room temperature for 1 h. Enhanced chemiluminescence (ECL) reagent (Thermo) was used for visualizing protein signals. Quantitation was performed using Image J software.

### Xenograft model

A total of 20 specific-pathogen-free (SPF) BALB/c nude mice (3–4 weeks old, female) were obtained from Shanghai SLAC Laboratory Animal Co., Ltd (Shanghai, China). Animal studies were approved by the Institutional Animal Care and Use Committee of Zhuzhou Central Hospital. They were maintained in a humidity-and temperature-controlled environment under pathogen-free condition, with a 12 h dark/light cycle. The animals had free access to food and water. Subsequently, mice were randomly divided into 4 groups (n = 5 per group). PC-3 cells (1 × 10^6^) were mixed with CAFs (1 × 10^6^) transfected with/without si-ATG5 or NFs (1 × 10^6^) in 200 µL Matrigel diluted with PBS (v/v = 1:1). Next, the flanks of mice were subcutaneously injected with the above mixed cells. Tumor growth was examined every 5 days. Mice were sacrificed at day 35 after tumor implantation. Tumor and lung samples were collected for the following research. Tumor volumes and weights were recorded. Tumor volume was calculated: Volume (mm^3^) = 0.5 × length × width^2^.

### Hematoxylin and eosin (H&E) staining

After fixed in 10% paraformaldehyde, lung tissues were dehydrated and embedded in paraffin. Then they were cut into slices (a thickness of 4 μm), followed by deparaffinized. Next, slices were stained by hematoxylin and then with eosin. A microscope (Olympus) was used for observing and photographing the samples. The number of lung metastatic nodules was then assessed.

### Immunohistochemistry

Tumor sections were deparaffinized and hydrated. Next, citrate buffer (0.01 M) was applied for antigen retrieval. After treated with 3% H_2_O_2_, slices were blocked at room temperature for 1 h by 10% goat serum. Slices were incubated at 4 °C overnight with primary antibodies, including anti-Vimentin (Abcam) and anti-Ki67 (Abcam). After that, secondary antibodies were added and incubated for 30 min at room temperature. Then 3,3′-diaminobenzidine (DAB) was used to visualize the immune complexes. Following counterstained with hematoxylin, slices were observed with a microscope (Olympus). Positive staining cells were counted on 5 randomly chosen visual fields by Image J.

### Statistical analysis

Data are described as mean ± standard deviation (SD). One-way ANOVA was employed to determine the differences among more than three groups. Difference between two groups was compared with Student’s t-test. p value < 0.05 was defined as statistical significance. Data were processed with SPSS software (IBM, Armonk, USA).

## Results

### Identification and autophagic status of CAFs

**Fig. 1:**
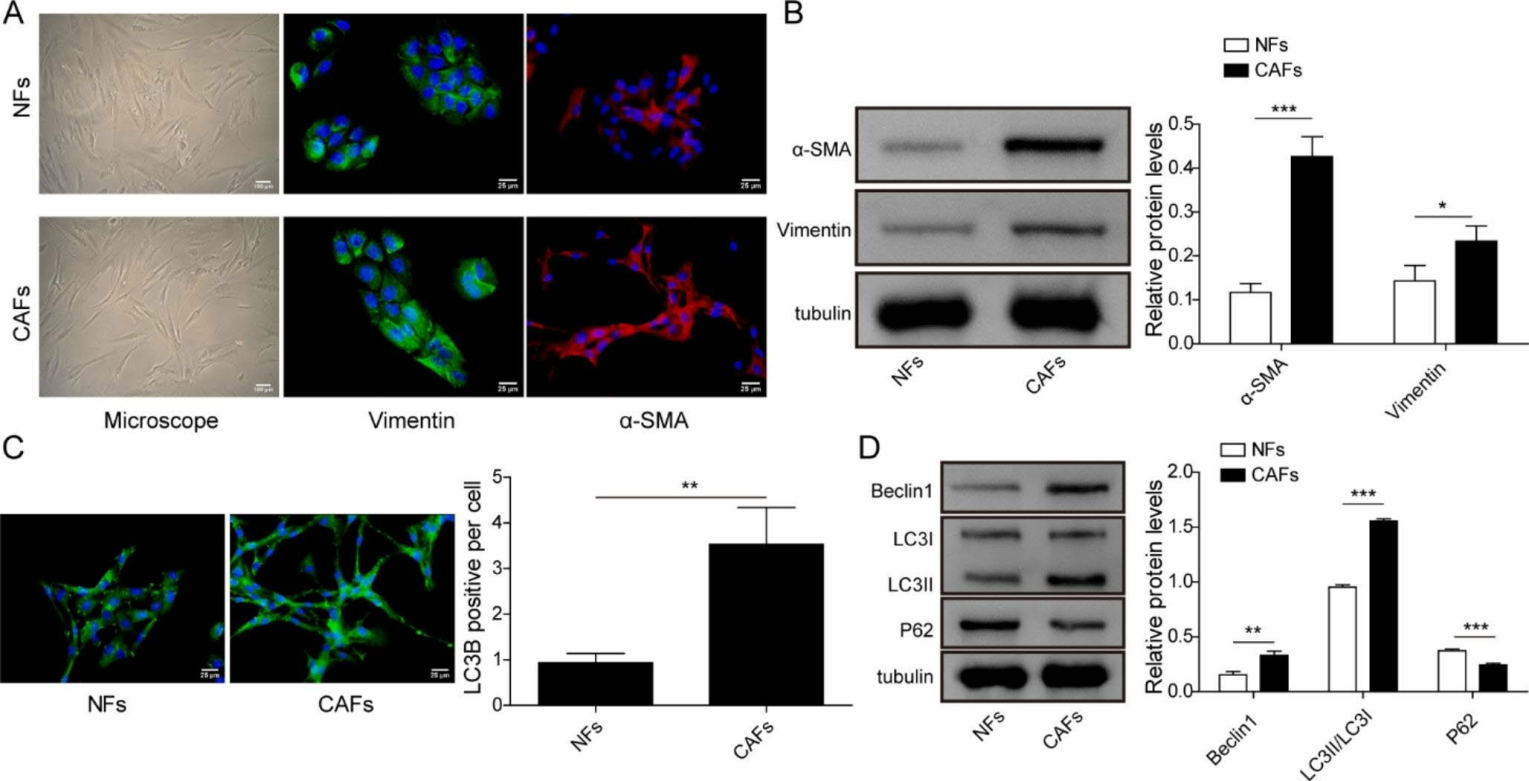
Identification and autophagic status of CAFs NFs and CAFs were isolated from adjacent normal tissues and cancer specimens, respectively. (A) Cell morphology and Vimentin and α-SMA immunofluorescence were observed by a microscope. (B) Western bolt was used to detect Vimentin and α-SMA levels. (C) The number of LC3B puncta detected by immunofluorescence. (D) Western bolt was utilized for detecting the expressions of autophagy-related proteins, including Beclin1, LC3II/LC3I ratio and p62. Data are presented as mean ± SD from three independent experiments. * p < 0.05, ** p < 0.01, *** p < 0.001

Firstly, we identified CAFs and NFs which were derived from PCa specimens and adjacent normal tissues, respectively. There was no obvious difference in morphology between CAFs and NFs; immunofluorescence and western blot showed that mesenchymal marker Vimentin and myofibroblast marker α-SMA were highly expressed in CAFs, compared to that in NFs **(**Fig. 1A, B**)**. We then evaluated the autophagic level in CAFs and NFs. The number of LC3B-positive puncta per cell in CAFs was more than that in NFs, suggesting the significant induction of autophagosome in CAFs **(**Fig. 1C**)**. Furthermore, Beclin1 and LC3II/I ratio, which represented autophagy level, and p62, a downstream protein modulated by LC3, were then measured using western blot. Beclin1 and LC3II/I ratio were higher, while p62 was lower in CAFs than that in NFs **(**Fig. 1D**)**. The data indicated that CAFs possessed a higher autophagic level.

### Weakened autophagic level of CAFs induced by 3-MA suppressed proliferation, migration and invasion of PCa cells

**Fig. 2:**
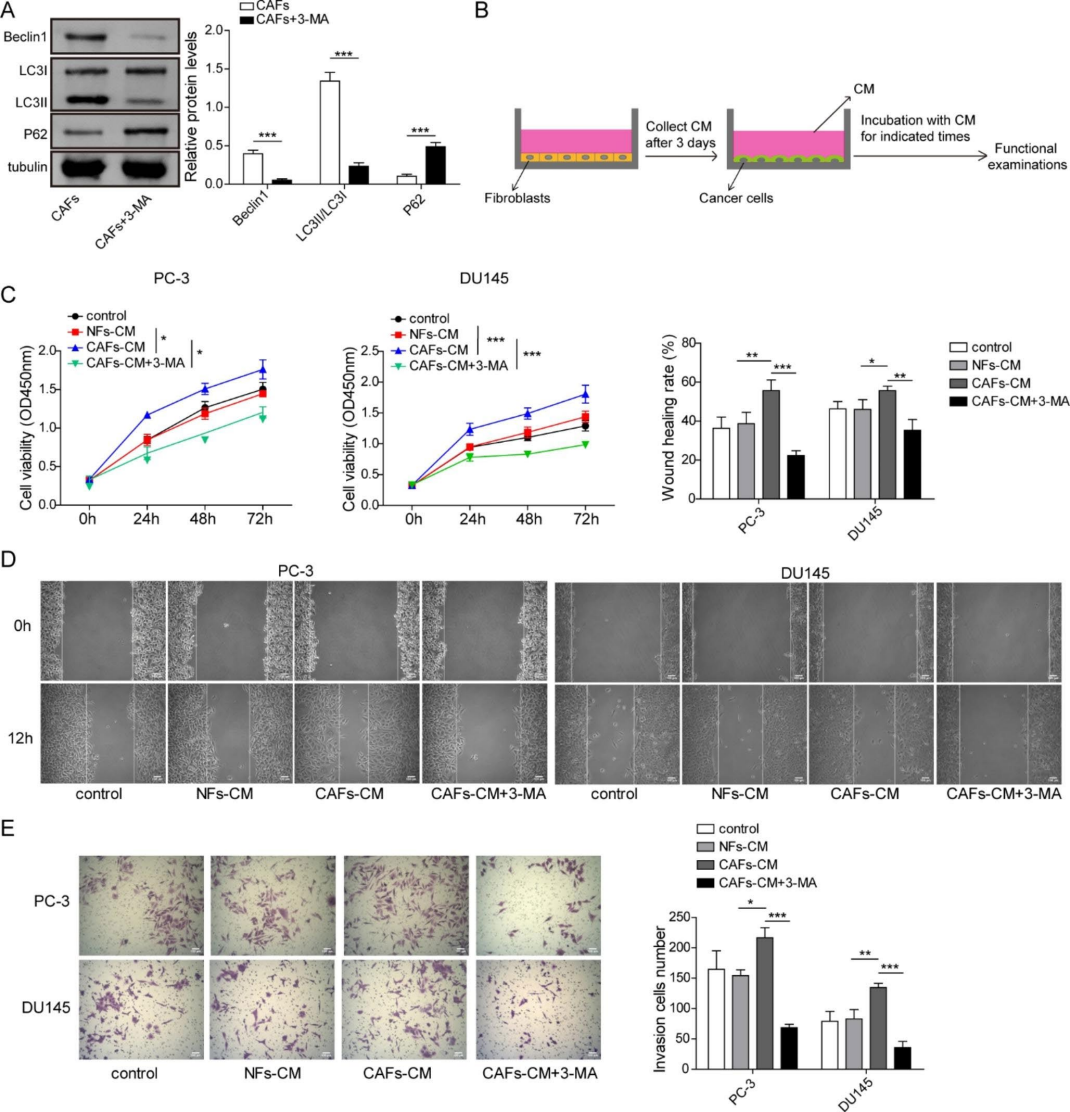
Weakened autophagic level of CAFs induced by 3-MA suppressed proliferation, migration and invasion of PCa cells CAFs were pretreated with autophagy inhibitor 3-MA. (A) Western bolt detection of autophagy- related proteins. (B-E) CM was collected from NFs or CAFs with or without 3-MA pretreatment, then PCa cells were incubated with NFs-CM or CAFs-CM. (B) The schematic illustration of CM collection and PCa cells incubation with CM. (C) Cell viability was evaluated using CCK-8 assay. (D) Representative pictures and the quantification of wound closure rates in wound healing assay. (E) Transwell assay for cell invasion measurement. Data are presented as mean ± SD from three independent experiments. * p < 0.05, ** p < 0.01, *** p < 0.001

To further investigate whether the higher autophagic level of CAFs affected the biological function of PCa cells, CAFs were pretreated with autophagy inhibitor 3-MA. Beclin1 and LC3II/I ratio were decreased while p62 was elevated in 3-MA-treated CAFs **(**Fig. 2A**)**, indicating that autophagic level in CAFs was expectedly repressed by 3-MA. Subsequently, PCa cells were incubated with CM derived from NFs or CAFs with or without 3-MA pretreatment **(**Fig. 2B**)**. Compared to that in control group, PCa cell viability in NFs-CM group showed no significant alteration; CAFs-CM promoted PCa cell viability, whereas suppression of autophagy restrained the promotive impact of CAFs on PCa cell viability **(**Fig. 2C**)**. PCa cells showed no significant difference in cell migration when they were incubated with NFs-CM; CAFs-CM accelerated PCa cell migration, however, inhibition of autophagy reversed the effect **(**Fig. 2D**)**. NFs-CM-treated cells showed no significant difference in cell invasion; however, CAFs-CM facilitated cell invasion, but the effect was overturned by inhibition of autophagy **(**Fig. 2E**)**. The data indicated that weakened autophagic level of CAFs repressed proliferation, migration and invasion of PCa cells.

### ATG5 served as an essential up-regulated gene for keeping autophagic status of CAFs

**Fig. 3:**
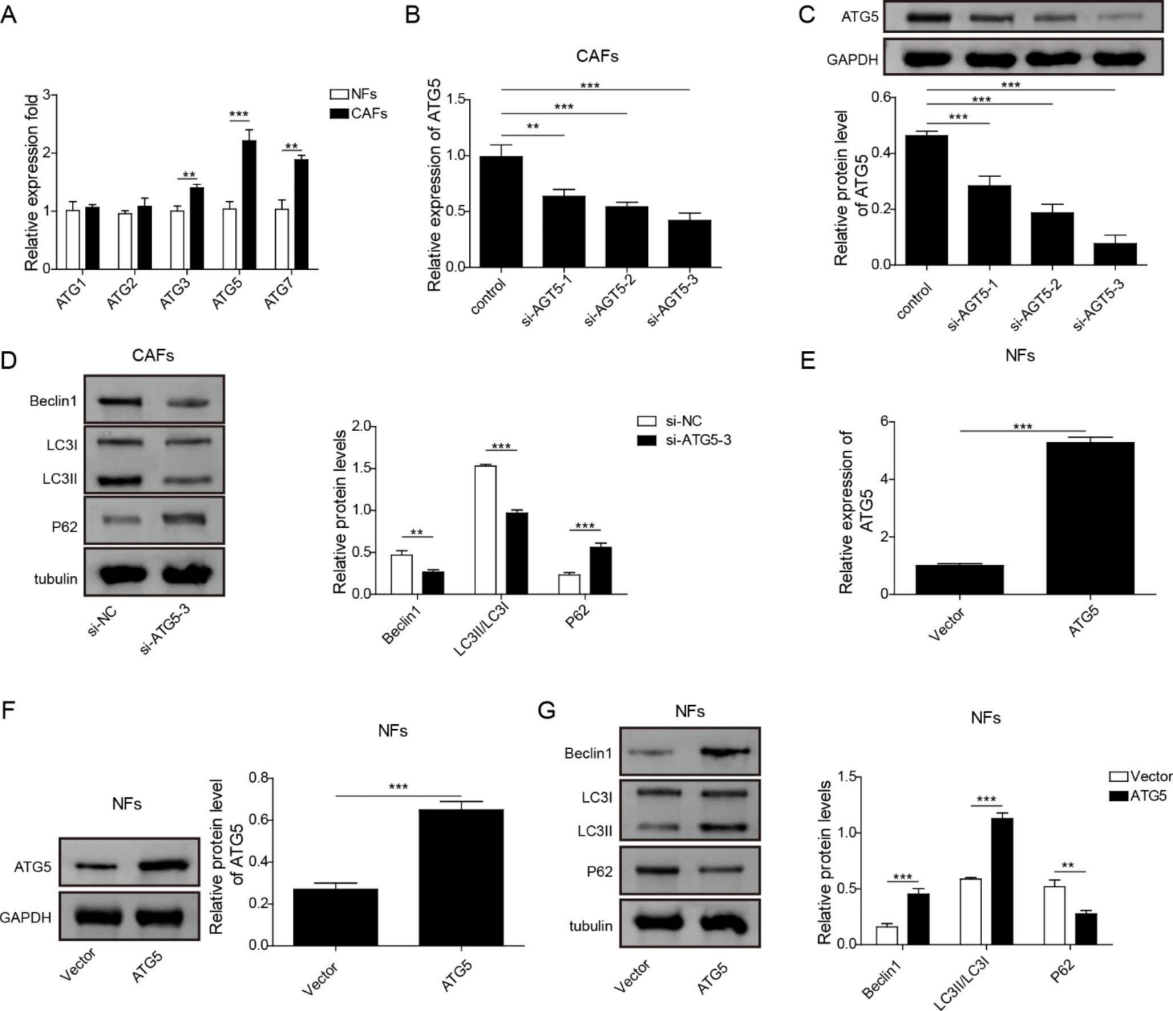
ATG5 served as an essential up-regulated gene for keeping autophagic status of CAFs (A) The expressions of ATGs (ATG1, ATG2, ATG3, ATG5 and ATG7) in NFs and CAFs were determined using RT-qPCR. (B, C) Three siRNAs specifically against ATG5 (si-ATG5-1, si-ATG5-2 and si-ATG5-3) were applied for cell transfection in CAFs, then the transfection efficiency was detected with RT-qPCR (B) and western bolt (C). (D) Western bolt was used to measure the expressions of autophagy-related proteins in CAFs transfected with si-ATG5-3 or si-NC. (E, F) ATG5 overexpression vector or control vector was transfected into NFs, and the transfection efficiency was determined with RT-qPCR (E) and western bolt (F). (G) Western bolt detected the expressions of autophagy-related proteins in NFs transfected with ATG5 overexpression vector or control vector. Data are presented as mean ± SD from three independent experiments. ** p < 0.01, ***p < 0.001

To investigate whether ATGs involved in the abnormal autophagic status of CAFs, endogenous ATGs mRNA levels in NFs and CAFs were then detected. Compared to that in NFs, ATG3, ATG5 and ATG7 mRNA levels were higher in CAFs **(**Fig. 3A**)**. Among them, ATG5 expression level was the highest in CAFs, which was selected for further investigation. To explore whether ATG5 affected the autophagic status of CAFs, we knocked down ATG5 level in CAFs by si-ATG5 transfection. The transfection efficiency of si-ATG5 was validated **(**Fig. 3B, C**)**. Due to the optimal transfection efficiency, Si-ATG5-3 was chosen for subsequent experiments. CAFs autophagic level could be inhibited by silencing of ATG5, indicated by the downregulated Beclin1 and LC3II/LC3I ratio and the upregulated p62 expression in CAFs **(**Fig. 3D**)**. Furthermore, we overexpressed ATG5 in NFs by transfection with ATG5 overexpression vector. The transfection efficiency of ATG5 overexpression vector in NFs were validated by RT-qPCR and western blot **(**Fig. 3E, F**)**. Overexpression of ATG5 could promoted NFs autophagic level, indicated by the elevated Beclin1 and LC3II/LC3I ratio and the declined p62 expression in NFs **(**Fig. 3G**)**. These data confirmed that ATG5 was a key gene upregulating autophagic level in CAFs.

### CAFs promoted proliferation, migration and invasion of PCa cells via ATG5

**Fig. 4:**
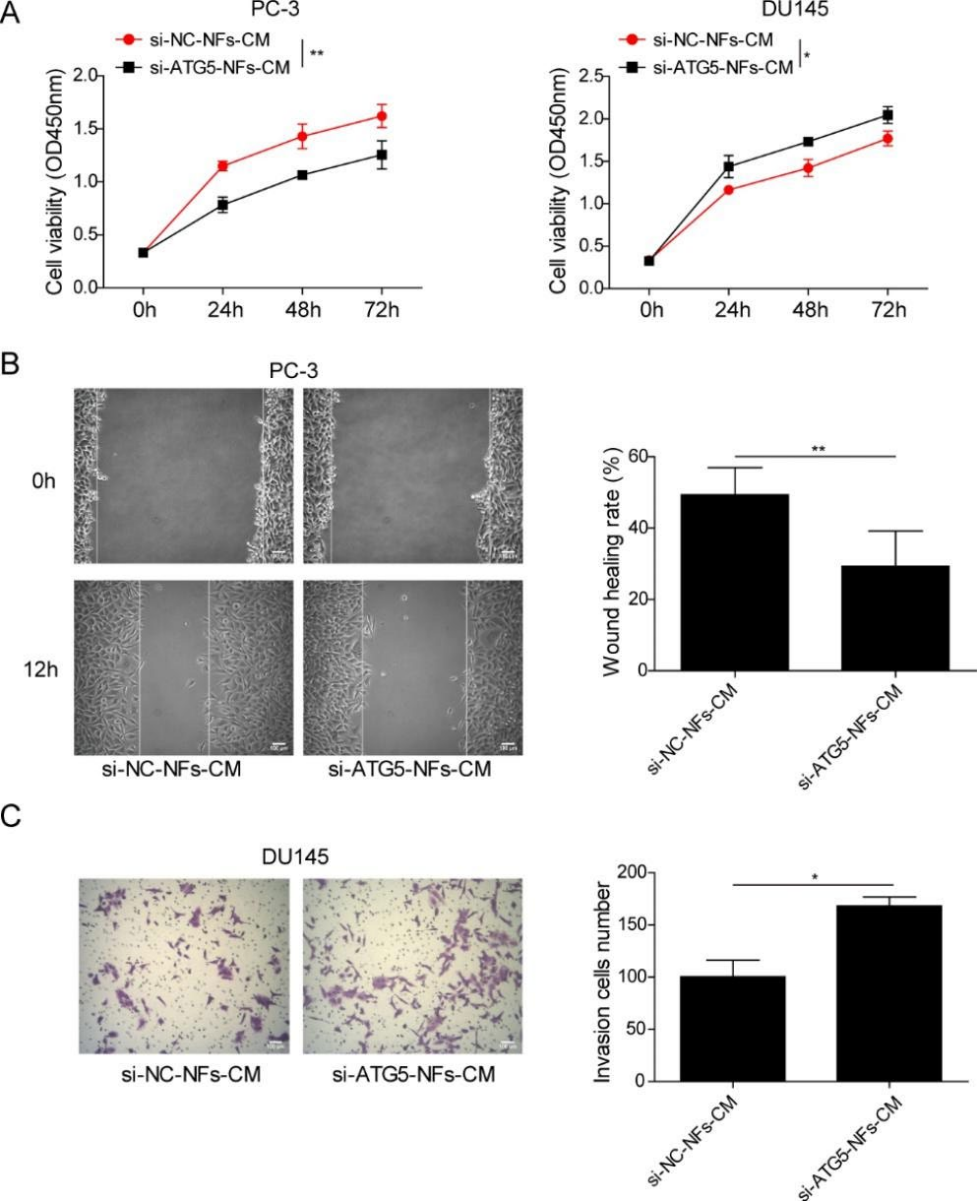
CAFs promoted PCa cells proliferation, migration and invasion via ATG5 (A-C) CAFs were transfected with si-NC or si-ATG5, besides, NFs were transfected with ATG5 overexpression vector or control vector. Then PCa cells were incubated with the CM from CAFs or NFs. (A) CCK-8 assay for cell viability measurement. (B) Wound healing assay for detecting cell migration. (C) Cell invasion assessed by Transwell assay. Data are presented as mean ± SD from three independent experiments. * p < 0.05, ** p < 0.01

We further studied whether ATG5 acted as a key gene to involve in CAFs’ effects on PCa cells. ATG5 was knocked down in CAFs by transfection with si-ATG5 or overexpressed in NFs by transfection with ATG5 overexpression vector, respectively. Then, PCa cells were incubated with the CM from CAFs or NFs. Compared to corresponding control groups, PCa cell viability was reduced by silencing of ATG5 in CAFs but elevated by overexpression of ATG5 in NFs **(**Fig. 4A**)**. Furthermore, knockdown of ATG5 in CAFs inhibited cell migration, whereas overexpression of ATG5 in NFs promoted cell migration **(**Fig. 4B**)**. Cell invasion of PCa cells presented the similar outcomes **(**Fig. 4C**)**. The above data confirmed that upregulated ATG5 in CAFs was responsible for CAFs’ promotive effects on PCa cell proliferation, migration and invasion.

### Depletion of ATG5 in CAFs inhibited xenograft tumor growth of PCa

**Fig. 5:**
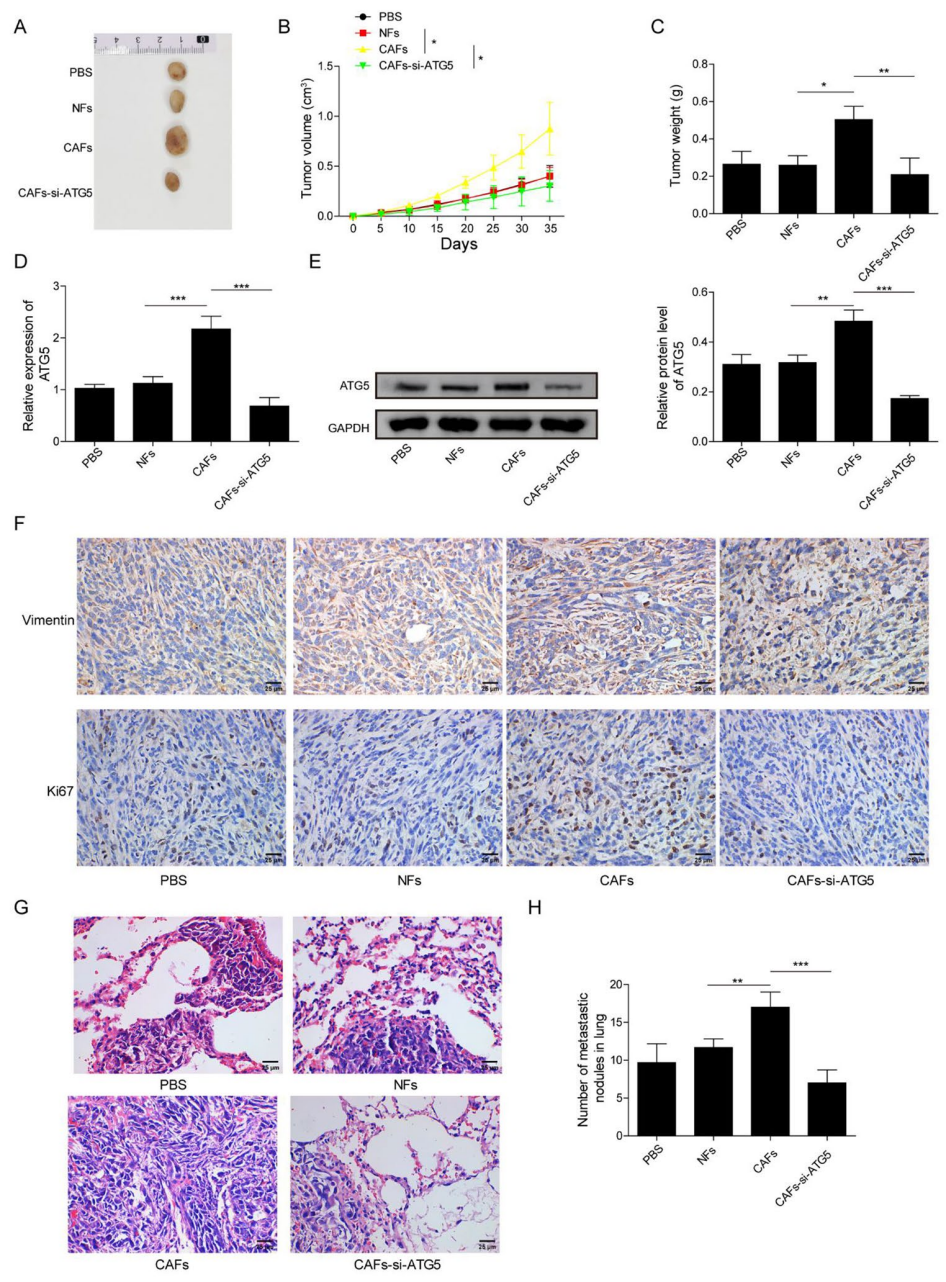
Depletion of ATG5 in CAFs inhibited xenograft tumor growth of PCa Nude mice were subcutaneously injected with PC-3 cells that mixed with NFs or CAFs transfected with si-ATG5 or not. (A-C) Xenograft tumor sizes (A), volumes (B) and weights (C). (D, E) RT-qPCR and western bolt for detecting ATG5 levels in tumor tissues. (F) Vimentin and Ki67 expressions in tumor tissues detected by immunohistochemistry. (G, H) The histopathological characteristics of lung tissues were evaluated by H&E staining. Lung metastatic nodules from different groups were counted. Data are presented as mean ± SD from three independent experiments. * p < 0.05, ** p < 0.01, ***p < 0.001

The in vivo experiment was conducted to further confirm the exact biological function of CAFs in PCa tumor growth. PC-3 cells were mixed with CAFs or NFs before injection into nude mice. Moreover, CAFs transfected with si-ATG5 were also employed to mix with PC-3 cells for illuminating the function of ATG5 in CAFs’ effects on tumor growth. Compared with that of PC-3 + PBS or PC-3 + NFs groups, CAFs could elevate tumor size, volume and weight, suggesting that CAFs accelerated xenograft tumor growth; whereas knockdown of ATG5 abrogated CAFs’ effects on tumor growth **(**Fig. 5A-C**)**. CAFs increased ATG5 mRNA and protein levels, and upregulated Vimentin and Ki67 expressions; however, CAFs-induced effects were attenuated after silencing of ATG5 **(**Fig. 5D-F**)**. The number of lung metastatic nodules in CAFs-treated mice was increased, which was reduced after knockdown of ATG5, confirmed by H&E staining **(**Fig. 5G, H**)**. Taken together, depletion of ATG5 in CAFs repressed PCa tumor growth.

## Discussion

PCa is one of the most diagnosed cancer types and a common causes of cancer-related deaths in men worldwide [26]. The metastatic PCa is still largely incurable even following intensive multimodal treatment, in spite of high long-term survival of localized PCa [27]. Recent studies have revealed that CAFs are critical for cancer progression [28]. Our study showed that PCa-derived CAFs possessed higher autophagic level, importantly, weakened autophagic level of CAFs induced by autophagy inhibitor suppressed PCa cells proliferation, migration and invasion. Moreover, this research demonstrated that CAFs promoted malignant phenotypes and tumor growth of PCa via ATG5.

TME represents the components and non-cancerous cells displayed in tumor, besides, the communication between TME and tumor cells plays a crucial role in the progression and metastasis of tumor [29]. Among TME, CAFs are the most abundant cellular components, and they are actively involved in cancer progression [30]. Recent studies show that autophagy is implicated in cancer development, moreover, suppression of autophagy may be an efficient therapeutic approach for advanced cancer [31]. The possible involvement of autophagy in PCa progression is indicated by the abnormal expressions of autophagy-related genes in PCa cells [32]. CAFs autophagy has been reported to be responsible for CAFs’ effect on tumor cells. For instance, CAFs autophagy can enhance breast cancer cell migration, invasion and proliferation [33]; CAFs regulate bladder cancer invasion through autophagy [34]. Furthermore, previous studies have demonstrated that CAFs can promote prostate tumor growth and progression [25, 35]. Therefore, considering that CAFs have been validated to be key regulators for PCa progression and autophagy is conservative for most cells, autophagic status of CAFs and its effects on PCa cells were investigated in our study. PCa-derived CAFs were discovered to present higher level of autophagy, as revealed by the elevated number of LC3B-positive puncta and the higher autophagy-related proteins (Beclin1 and LC3II/LC3I ratio). Inhibition of autophagy by 3-MA mitigated CAFs’ impacts on PCa cells proliferation, migration and invasion. Our study revealed that autophagy activity of CAFs affected the development of PCa. Collectively, our results provided evidence showing that autophagy promotion is a potential mechanism underlying PCa progression induced by CAFs. CAFs can promote PCa progression through growth factor-beta (TGF-β) pathway [25], upregulation of cholesterol and steroid biosynthesis [35] and loss of Caveolin-1 in CAFs [36]. Our study indicates a novel mechanism that higher autophagy level of CAFs contributes to CAFs’ effects on PCa progression.

ATG5-dependent autophagy is involved in the development of PCa, for instance, upregulating ATG5 and autophagy level promotes proliferation and migration in PCa cells [37]. Besides, downregulation of ATG5 inhibits autophagy and suppresses migration and invasion in PCa cells [38]. We demonstrated that ATG5 served as an essential up-regulated gene for keeping autophagic status of CAFs, as indicated by the evidence that silencing of ATG5 in CAFs inhibited CAFs autophagic level, while overexpression of ATG5 in NFs promoted NFs autophagic level. Furthermore, when ATG5 knockdown inhibits CAFs autophagy, invasion of non-small cell lung cancer cells can be repressed [24]. We reported that upregulated ATG5 expression in CAFs was responsible for CAFs’ promotive effect on malignant phenotypes of PCa cells. Additionally, our in vivo study revealed that ATG5 silencing could diminish CAFs’ promotive effects on xenograft tumor growth and lung metastasis. This research indicated that high level of ATG5 in CAFs resulted in the enhanced autophagy activity, thereby facilitating PCa progression.

PC-3 and DU145 cell lines are androgen-insensitive cells and applied as representatives of castration-resistant prostate cancer [39–41]. Further studies are needed to carry out in PCa cell lines that express wild-type androgen receptor, to explore the role of CAFs autophagy in PCa progression. Moreover, PCa is also sensitive to estrogens [42]. Prostate CAFs display low estrogen receptor (ER)-α and high G protein-coupled receptor 30 (GPR30); besides, GPR30 overexpression or ER knockdown induces CAF-like phenotype of prostate stromal cells and promotes proliferation and migration of PCa cells [43]. The involvement of estrogen receptors in CAFs autophagy warrants further investigation in PCa. It has been reported that CM from human PCa PC-3 cells has the capacity to induce a CAF-like phenotype in human prostate stromal cells [44]. Our present study only focuses on the impact of CAFs or NFs on PCa cells, whereas we have not explored the influence of PCa CM on NFs or CAFs.

## Conclusion

In summary, PCa-derived CAFs possessed higher autophagic level. Importantly, accompanied by the aberrantly upregulated ATF5, CAFs exhibited a promotive action on malignant phenotypes of PCa cells through autophagy. This study improved our understanding of the mechanism underlying CAFs-mediated promotive effects on PCa progression and might provide a novel target for PCa treatment.

## Electronic supplementary material

Below is the link to the electronic supplementary material.

Supplementary Material 1

## Data Availability

All data generated or analyzed during this study are included in this published article.

## Abbreviations

CAFs: Cancer-associated fibroblasts
NFs: Normal fibroblasts
PCa: Prostate cancer
CM: Conditioned medium
α-SMA: α-smooth muscle actin
3-MA: 3-Methyladenine
TME: Tumor microenvironment
ATG: Autophagy-related genes
ATG5: Autophagy-related 5
TGF-β: Transforming growth factor-beta
ER: Estrogen receptor
GPR30: G protein-coupled receptor 30
