# Supplementary material for: Cancer-associated fibroblasts promote malignant phenotypes of prostate cancer cells via autophagy: Cancer-associated fibroblasts promote prostate cancer development
Source: Apoptosis. 2023 Mar 31;28(5-6):881–91. doi: 10.1007/s10495-023-01828-2 (PMC10232635; doi:10.1007/s10495-023-01828-2)
Supplement: Supplementary file 1 — Supplementary Material 1 [file 10495_2023_1828_MOESM1_ESM.docx]

**Table 1.** **Clinical and pathologic characteristics of patients.**

| Parameter | **Range** | **Tumor stage (number of patients)** | | | |
| --- | --- | --- | --- | --- | --- |
|  |  | pT1-2 | pT3-4 | N1 | Sum |
| Number of patients |  | 2 | 2 | 1 | 5 |
| Age(years) | ≤60 | 0 | 1 | 0 | 1 |
|  | ＞60 | 2 | 1 | 1 | 4 |
| Tumor grade (Gleason score) | ≤7 | 1 | 0 | 0 | 1 |
|  | ＞7 | 1 | 2 | 1 | 4 |
| Serum PSA (ng/mL) | ≤10 | 1 | 0 | 0 | 1 |
|  | ＞10 | 1 | 2 | 1 | 4 |

**Table 2. The primer sequences used in RT-qPCR.**

| Gene | Forward primer (5’-3’) | Reversed primer (5’-3’) |
| --- | --- | --- |
| ATG1 | TTAACCGCTCGGCTCTGATTTC | AAGCTCCTTTATGAGATGCTCGATTC |
| ATG2 | GATTTCGATACAATGGCATTTTG | ACCCTATAGAAACGTCCAAGTTAG |
| ATG3 | GATGGCGGATGGGTAGATACA | TCTTCACATAGTGCTGAGCAATC |
| ATG5 | GTTTTGGGCCATCAATCGGAA | TCTCCTAGTGTGTGCAACTGT |
| ATG7 | ATGATCCCTGTAACTTAGCCCA | CACGGAAGCAAACAACTTCAAC |
| GAPDH | AGCCACATCGCTCAGACAC | GCCCAATACGACCAAATCC |
